# Role of Genetic Interactions in Alzheimer’s Disease: Lessons from Long Life Family Study (LLFS)

**DOI:** 10.1093/geroni/igaa057.1589

**Published:** 2020-12-16

**Authors:** Anatoliy Yashin, Dequing Wu, Konstantin Arbeev, Eric Stallard, Qihua Tan, Alexander Kulminski, Mary Feitosa, Svetlana Ukraintseva

**Affiliations:** 1 Duke University, Durham, North Carolina, United States; 2 Duke University, Durham, United States; 3 Duke University, Biodemography of Aging Research Unit, Durham, North Carolina, United States; 4 University of Southern Denmark, Odense C, Denmark; 5 Washington University School of Medicine, St Louis, Missouri, United States

## Abstract

Experimental and clinical studies of Alzheimer’s disease (AD) provide plentiful evidence of AD heterogeneity and involvement of many interacting genes and pathways in regulation of AD-related traits. However, detailed mechanisms of genetic interactions (GxG) involved in AD remain largely unknown. Uncovering hidden patterns of such interactions from human data will help better understand the nature of AD heterogeneity and find new targets for AD prevention. In this paper, we applied a newly developed method of evaluating joint GxG effects on AD to analysis of the Long Life Family Study data. The analysis included several steps: (i) selecting candidate genes from stress response pathways that are thought to be involved in AD; (ii) estimating interaction effects of SNP-pairs on AD risk, and selecting the top interacting SNPs; (iii) running GWAS-like interaction analysis for SNP-pairs, with one SNP fixed; (iv) using characteristics of the detected SNP-pairs interactions to construct the SNP-specific Interaction Polygenic Risk Scores (IPRS); and (v) evaluating the effects of IPRSs on AD. We found that SNP-specific IPRS have highly significant effects on AD risk. For most SNPs involved in the significant interaction effects on AD, their individual effects were statistically not significant. Male and female analyses yielded different subsets of the top interacting SNPs. These results support major role of genetic interactions in heterogeneity of AD, and indicate that AD mechanisms can involve different combinations of the interacting genetic variants in males and females, which may point to different pathways of resistance/response to stressors in two genders.

